# Combined application of straw and biochar: reducing nitrogen fertilizer demand and enhancing soybean yield

**DOI:** 10.3389/fpls.2026.1839497

**Published:** 2026-05-28

**Authors:** Meng Li, Tao Du, Qiuju Wang, Xiaofang Wang, Saqib Saleem Akhtar, Sophie Minori Uchimiya, Sven-Erik Jacobsen, Mo Li, Aizheng Yang

**Affiliations:** 1 Key Laboratory of Coupling Process and Effect of Natural Resources Elements, Beijing, China; 2 College of Hydraulic Science and Engineering, Northeast Agricultural University, Harbin, Heilongjiang, China; 3 Chinese Hydraulic Engineering Society, Beijing, China; 4 Heilongjiang Province Black Soil Protection and Utilization Research Institute, Harbin, Heilongjiang, China; 5 Roskilde Agro ApS, Gadstrup, Denmark; 6 USDA-ARS Southern Regional Research Center, New Orleans, LA, United States; 7 Quinoa Quality ApS, Regstrup, Denmark

**Keywords:** biochar, grain yield, nitrogen regulation, soil hydrothermal environment, soil nutrients, straw

## Abstract

Soybean (*Glycine max* (L.) Merr.) is an important food crop in Northeast China. However, intensive cultivation relying on long-term high nitrogen fertilizer input has led to the loss of soil organic carbon and the decline of cultivated land quality in the black soil region, compromising soil sustainability. Therefore, it is urgent to explore a cultivation mode that achieves both stable soybean yield and coordinated improvement of soil sustainability. The application of biochar and straw return presents a promising strategy to enhance crop productivity and agricultural sustainability. However, the mechanisms underlying these improvements, particularly those driven by the combined application of straw and biochar under reduced nitrogen application, remain unclear. From 2022 to 2023, a field experiment was conducted on black-calcium soil using ‘Dongnong 55’ soybeans. Two biomass-return methods were evaluated: straw addition and a straw-biochar mixture addition, under six treatments: CK (conventional N with full straw return), SN2 (80% N with full straw), SN3 (60% N with full straw), SBN1 (conventional N with straw - biochar mix), SBN2 (80% N with straw - biochar mix), and SBN3 (60% N with straw - biochar mix). The results revealed that straw–biochar co-application improved the soil hydrothermal environment and nutrient supply compared with straw return alone. In the 0–20 cm soil layer, soil moisture content increased by 9.0% - 14.3%, while available N, P, and K increased by 47.5%–48.9%, 56.8%–71.7%, and 28.4%–41.2%, respectively. These improvements enhanced soybean physiological performance, increasing chlorophyll content, net photosynthetic rate, and leaf intrinsic water use efficiency by 12.3%, 20.6%, and 16.9%, respectively. Notably, SBN2 increased soybean yield by 9.3%–11.0% compared with SN2, and maintained a yield statistically comparable to SBN1 despite a 20% reduction in N input. Therefore, SBN2 was identified as the optimal treatment because it maintained soybean yield while reducing N fertilizer input and improving soil water, heat, nutrient supply, and resource-use efficiency. This study provides a valuable reference for managing agricultural straw, biochar utilization, and nitrogen fertilizer.

## 1 Introduction

Soil plays an important role in terrestrial ecosystem, performing important functions like water retention, nutrient cycling and crop production (Cotrufo et al., 2011). The soil texture of the black soil region of China is relatively clay - rich and dense, primarily silty clay or clay loam, which is recognized as one of the most vital soil resources due to its high content of soil organic carbon (SOC) and total nitrogen (TN) (Wei et al., 2008). However, rapid population growth and increasing food demand have led to inconsistent farming practices, including inappropriate nitrogen application and intensive cultivation, aimed at maximizing crop yields. These practices have significantly degraded black soil areas, posing serious threats to agricultural sustainability and ecosystem health (Bai et al., 2018). Therefore, to address this degradation, effective strategies for improving black soil quality and maintaining ecosystem functions are urgently needed. Straw return and biochar application are two promising approaches that have been shown to enhance soil quality and promote crop growth (Liu et al., 2022a). However, most research has focused on the independent application of straw or biochar, with limited attention to their combined effects on soil nutrients and crop performance.

Crop yield exhibits an inverted U-shaped curve with increasing nitrogen fertilizer input, and continuous increase in nitrogen fertilizer input has not resulted in a sustained increase in crop yield (Qiang et al., 2023). In contrast, the excessive application of chemical nitrogen fertilizer alters the nitrogen balance in the soil, leading to significant nitrogen loss and causing a series of environmental problems such as surface water eutrophication, groundwater nitrate accumulation, and greenhouse gas emissions (Wen et al., 2017). Therefore, it is imperative to identify sustainable strategies that maintain or enhance crop yields while avoiding further increases in nitrogen fertilizer inputs.

Crop straw is an important biomass resource generated in the process of agricultural production. In China, the amount of crop straw increased significantly rising from 382 million tons in 2003 to 657 million tons in 2022 (Liu et al., 2022b). However, the indiscriminate disposal of straw not only a source environmental pollution but also leads to large losses of soil organic matter and ultimately causes soil degradation (Kumar et al., 2019). Straw returning is one of the best ways to handle straw resources. Wu et al. (2022) reported that straw return can improve the soil structure and soil fertility which also reduce the nitrogen fertilizer needs from 10% to 20%. A meta-analysis by Ul Islam et al. (2022) showed that in dry land soils in China, straw return can increase crop yields by 6 to 12%. In a study of a rice–wheat rotation system with continuous fertilizer application over 3 years, Zhu et al. (2015) reported that a 50% increase in straw returning per year represented the optimal rate of straw returning, which significantly increased soil organic carbon sequestration and grain yield. However, there are several negative effects of straw returning. In the early stages of straw decomposition, the effective nitrogen in the soil is depleted, decreasing the nitrogen available for crop growth and thus reducing food production. In addition, it takes approximately 3 years for straw to fully decompose into nutrients that can be utilized directly by crops (Alvaro-Fuentes et al., 2013). Simultaneously, straw can carry numerous germs and insect eggs. Once straw is returned to the field, these germs and eggs are buried in the soil, providing an amenable environment for the breeding of pests and diseases (Shan et al., 2021). Additionally, microorganisms in the soil will multiply and decompose the organic matter in the straw, leading to an elevation of the soil carbon to nitrogen ratio, which in turn decreases the availability of nitrogen in the soil (Zhao et al., 2019). This causes the microorganisms in the soil to compete with crops for nitrogen, resulting in nitrogen deficiency during the early growth stages of crops (Ouyang et al., 2016). An in-depth discussion of the efficient use of straw resources to increase soil quality, improve the soil nutrient supply and increase crop yields is needed.

In addition to the direct incorporation of straw back into the field, it may also undergo transformation into biochar. Biochar is a stable, carbon-rich material formed via the pyrolysis of biomass (including forestry and agricultural residues) under low-oxygen conditions (Thi et al., 2017). Biochar is beneficial for improving the soil nutrient status and increasing crop yields owing to abundant micropores, high specific surface area, strong adsorption capacity and high nutrient content (Bass et al., 2016). For instance, biochar incorporation into fields can result in increased organic carbon, moisture and nutrient contents in soils (Sadaf et al., 2017). Ippolito et al. (2016) reported that biochar increased the TN and K contents of soil, promoted rice growth, and increased rice yield. He et al. (2020) demonstrated that biochar promoted N retention and adsorption and significantly improved the photosynthetic capacity of crops. However, excessive application of biochar to soils could also have some negative impacts. Jeffery et al. (2011) demonstrated that in certain soils, when the application rate of biochar exceeds a specific limit (e.g., 5 - 10%), it can adsorb a substantial amount of ammonium - nitrogen, thereby reducing the quantity of nitrogen accessible for plant absorption. Borchard et al. (2019) found that when the application amount of biochar exceeded 20% of the soil mass, the soil’s aeration and water permeability were reduced, thereby having a negative impact on plant growth. Additionally, the production of biochar is not only costly but also energy-intensive, significantly limiting its widespread adoption and application. Consequently, an integrated application of biochar and straw presents a feasible strategy for enhancing soil fertility, quality, crop development, and overall yield.

Previous studies have predominantly focused on either straw return or biochar application individually, with limited research addressing the combined effects of straw and biochar application on soil nutrients and crop growth in black soil regions under varying nitrogen application rates. The study aimed to investigate the effects of the combined application of straw and biochar under reduced nitrogen fertilization on (1) soil hydrothermal properties in different soil layers during various stages soybean growth; (2) the dynamics of soil nutrients, including SOC, TN, available nitrogen (AN), total potassium (TK), available potassium (AK), total phosphorus (TP) and available phosphorus (AP); (3) the growth indices and yield components throughout key developmental stages. Furthermore, this research sought to elucidate the mechanisms by which the combined application of straw and biochar affects soil nutrients, soybean growth and yield. The findings provide critical insights into the sustainable and efficient use of agricultural straw and biochar and offer guidance for optimizing nitrogen fertilizer management in black soil regions.

## 2 Materials and methods

### 2.1 Description of the experimental site and samples

**Figure 1:**
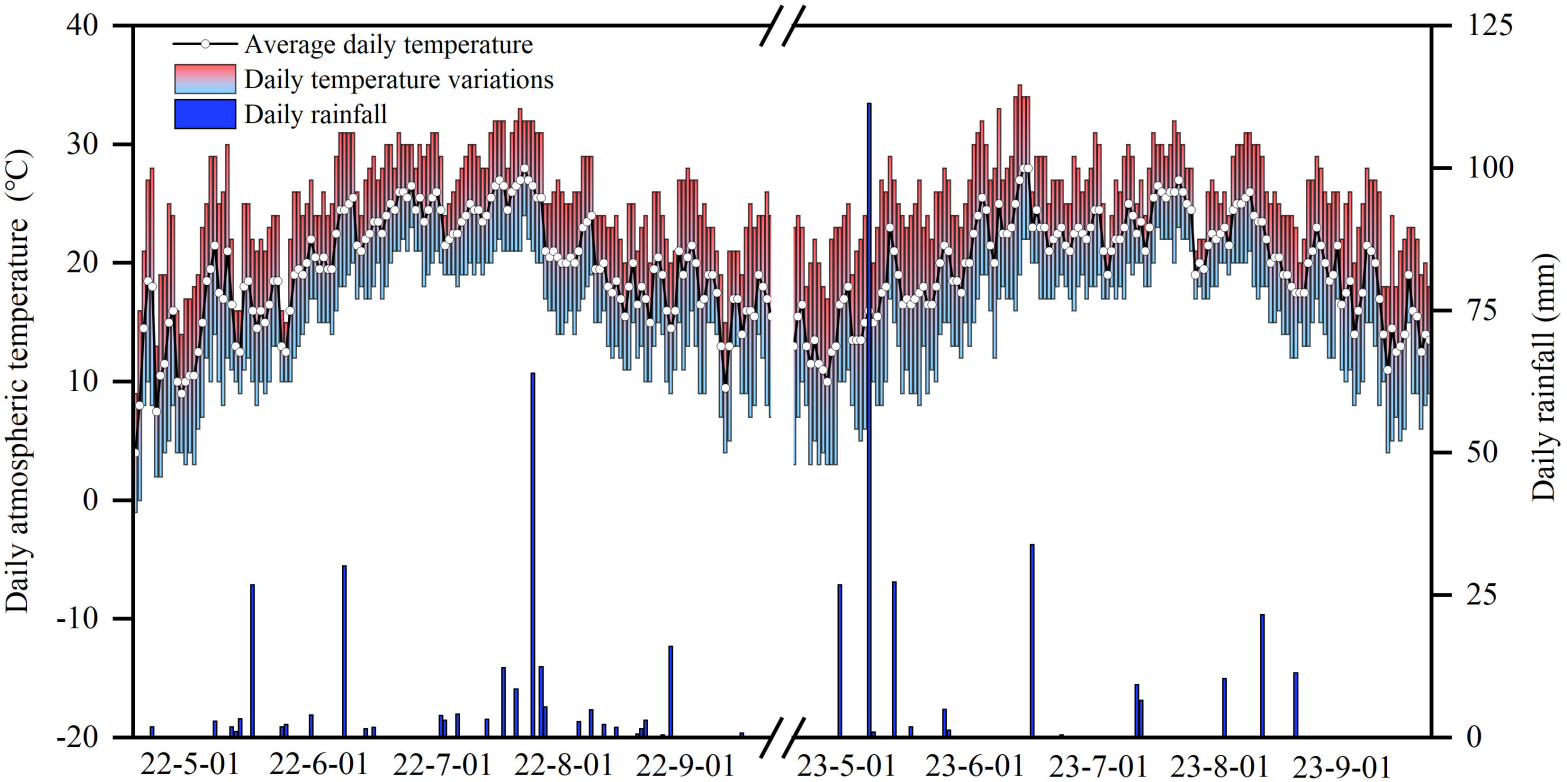
Dynamics of daily rainfall and temperature during the 2022–2023 soybean cropping cycle.

The experimental site was in the Acheng Experimental Demonstration Base of Northeast Agricultural University of Heilongjiang, located in Harbin city, Heilongjiang Province (latitude 45°01′N, longitude 126°02′E, mean elevation 162 m), where there is a temperate continental monsoon climate with obvious seasonal variations. The long-term average annual rainfall is 563.2 mm, with an evaporation volume of 796 mm. The average annual temperature is 3.4 °C, and the frost-free period lasts for 142 days. The daily rainfall and temperature during the experimental periods are shown in **Figure 1**. The soil in the study area is black soil with a silty clay texture (Lu et al., 2008), and its basic physical and chemical properties are summarized in **Table 1**. The basic physicochemical properties of the straw used in the experiment are shown in **Table 2**. The biochar used in this experiment was made from corn stover pyrolyzed at 500 °C under anaerobic conditions. The basic physicochemical properties of the biochar are shown in **Table 3**. The data were extracted from the monthly data of Harbin weather station. (https://www.tianqi24.com/haerbin/).

**Table 1:** Basic properties of the test soil.

Soil depth (cm)	Bulk density (g cm^-3^)	TN (g kg^-1^)	SOC (g kg^-1^)	AN (mg kg^-1^)	AP (mg kg^-1^)	AK (mg kg^-1^)
0-20	1.4	0.7	10.5	38.5	18.6	117.5
20-40	1.6	0.6	10.1	35.4	15.3	108.4

**Table 2:** Basic physical and chemical properties of straw.

Densities (g/cm³)	pH	C/N	N (%)	P (%)	K (%)
0.1	5.8	78/1	0.9	0.2	1.3

**Table 3:** Basic physicochemical properties of the biochar.

Grain size (mm)	Carbon content (%)	Total nitrogen (%)	Sulphur mass fraction (%)	Hydrogen mass fraction (%)	Ash (%)
1.5-2.0	70.4	1.5	0.8	1.7	31.8

### 2.2 Experimental design

The field experiment was conducted during two consecutive soybean growing seasons, from May to September in 2022 and 2023. One soybean crop was grown each year, and no other crop was planted during the experimental growing season. The experiment included two treatment factors: the main factor was field material, including full straw return (S), representing conventional straw utilization methods in the region, and the combined application of straw and biochar (SB). The second factor was N application rates, including 56.3 kg N ha^-1^ (conventional N application in the region, N1), 45.0 kg N ha^-1^ (80% N application, N2) and 33.8 kg N ha^-1^ (60% N application, N3). The straw used for field return was maize stover, which represents the major crop residue resource in the local maize-soybean production region. In this region, the maximum yield of corn stover per crop season is about 40 t ha^-1^, and the conversion rate of stover to biochar is 30%. Half of the straw was used for biochar production. Consequently, the SB combination yielded 6 t ha^-^¹ of biochar after pyrolysis (**Table 4**). The experiment spanned two years, with identical treatments applied each year. Furthermore, the SN1 was designated as the control treatment (CK) for comparison. To account for production costs and potential negative effects of excessive biochar application on soil health and crop performance, a treatment involving the conversion of all straw to biochar and subsequent field return was excluded. The experimental design is shown in **Table 4**. Each treatment was replicated three times. The experimental plot was 40 m^2^ (5 m × 8 m) in size, with a 1–2 m buffer zone between plots to avoid boundary effects. Soybean planting involved 60 cm spacing between rows and 10 cm intervals between planting holes within rows, with two plants per hole. The plots were plowed consistently in both years: maize stover was incorporated into the 20–40 cm soil layer using a deep-tilling plow, while biochar was incorporated into the 0–20 cm soil layer using a rotary tiller. Soybean seeds (cv. ‘Dongnong 55’) were sown on 10 May 2022 and 11 May 2023, respectively. Plant sampling occurred at four key growth stages: seedling, flowering, bulging, and ripening. All fertilizers were applied once as basal fertilizer before sowing. Nitrogen was applied as urea (46% N), phosphorus was applied as calcium superphosphate (12% P_25_), and potassium was applied as potassium sulfate (50% K_2_O). The N application rates were 56.3, 45.0, and 33.8 kg N ha^-^¹ for N1, N2, and N3, respectively. Phosphorus and potassium fertilizers were applied at the same rates across all treatments, corresponding to 60 kg P_25_ ha^-^¹ and 45 kg K_2_O ha^-^¹, respectively. Pest management and other agronomic practices adhered to local standards.

**Table 4:** Utilization of straw, biochar, and nitrogen fertilizer across treatments.

Treatment	Straw usage (t ha^-1^)	Biochar usage (t ha^-1^)	Nitrogen fertilizer usage (kg ha^-1^)
SBN1	20	6	56.2
SBN2	20	6	45.0
SBN3	20	6	33.8
SN1(CK)	40	0	56.2
SN2	40	0	45.0
SN3	40	0	33.8

In the SB treatments, 20 t ha^-^¹ maize stover was directly returned to the field, while another 20 t ha^-^¹ maize stover was converted into 6 t ha^-^¹ biochar based on an approximate conversion rate of 30%.

### 2.3 Sample collection and analysis

#### 2.3.1 Soil moisture content and temperature

Soil temperature and soil moisture content were measured with an automatic ET100-type soil water and heat monitoring system. The system provided real-time monitoring of the soil temperature and moisture changes in the field. The monitoring period ranged from pre-sowing to postharvest every 30 minutes and the data was collected automatically by the data collector system. Instrument probes were placed in the middle of the test area. Measurements were taken at depths of 20, and 40 centimeters. Prior to field installation, the ET100 soil water probes were calibrated using soil samples collected from the experimental site at 0–20 and 20–40 cm depths. The sensor readings were compared with gravimetric soil water content measured by the oven-drying method, and calibration equations were established and applied to correct the monitored soil moisture data. During the growing season, additional soil samples were collected periodically near the probes to verify the sensor readings.

Equation 1 was used to calculate the accumulated temperature:

(1) $$Ta=\overset{\text{n}}{\underset{\text{i}=1}{\text{∑}}}T_{i}$$

where *Ta* is the accumulated temperature, °C; *Ti* is the average soil temperature from 0 to 40 cm on day *i*; and n is the number of days in the soybean fertility period, d. The average temperature of the soil on day i is the average temperature of the soil from 0 to 40 cm.

Equation 2 calculates agricultural water consumption (AWC) was calculated via the water balance method:

(2) $$\begin{array}{c} AWC=\Delta W+P+I+V-D-R \end{array}$$

where *AWC* is the agricultural water consumption during a given growth period (mm); *ΔW* is the change in soil water storage between the beginning and the end of the growth period (mm). It was determined as the difference between soil water storage at the beginning and at the end of each growth period within the 0–40 cm soil profile. Soil water storage was calculated from the volumetric soil water content and the thickness of each measured soil layer; *P* is the precipitation during the corresponding growth period (mm); *I* is the irrigation amount (mm); *V* is the groundwater recharge or upward capillary rise (mm); D is the deep drainage below the measured soil layer (mm); and *R* is the surface runoff (mm). As this was a rainfed experiment conducted on flat terrain with a deep groundwater table, *I* was set to 0, while *V*, *D*, and *R* were assumed to be negligible in the water balance calculation.

#### 2.3.2 Soil sampling

Soil samples were collected at two depths (0–20 cm and 20–40 cm) using a 5 cm diameter auger during four key growth stages of soybean in 2022 and 2023. Each soil sample was air-dried, and visible gravel, plant roots, and debris were removed and discarded. The soil samples were then ground, passed through a 2.0 mm sieve, and analyzed for soil organic carbon (SOC), total nitrogen (TN), available nitrogen (AN), total phosphorus (TP), available phosphorus (AP), total potassium (TK), and available potassium (AK) contents.

The physical and chemical properties of the soil were measured three times as described by Bao (2000). The SOC was determined by external heating with potassium dichromate and concentrated sulfuric acid. The TN content was determined via H_2_SO_4_ boiling and the Kjeldahl nitrogen determination method, whereas AN was measured using alkali hydrolysis diffusion. The TP content was determined via H_2_SO_4_-HClO_4_ acid solution-molybdenum antimony colorimetry, and the AP content was determined via sodium bicarbonate-molybdenum antimony anti colorimetry. The TK was measured via atomic absorption flame photometry, whereas the AK was determined via ammonium acetate extraction followed by flame spectrophotometry.

#### 2.3.3 Plant height, stem thickness and leaf area-particularly

Plant height, stem thickness, and leaf area were measured at the seedling, flowering, and bulging stages. In each plot, 10 representative plants were randomly selected for measurement. Plant height was measured from the soil surface to the top of the main stem using a ruler. Stem thickness was measured 1 cm below the cotyledonary node using a digital caliper. Leaf area was estimated using the length–width method, and the area of each leaf was calculated as leaf length × leaf width × 0.75.

#### 2.3.4 Plant photosynthesis, SPAD values and yield

Crop photosynthesis characteristics were measured via a photosynthesis meter (Li-6400, USA) from 10:00 am to 2:00 pm before the plants were collected for sampling. Functional leaves from the same part of each plant were used for the measurement of the net photosynthetic rate (Pn) and stomatal conductance (Gs). The relative leaf green content was determined with a chlorophyll meter (PhotosynQ LLC).

Equation 3 calculates leaf intrinsic water use efficiency (WUE_n_):

(3) $$WUEn=\frac{Pn}{Gs}$$

Yield components were measured at the maturity stage. In each plot, 10 representative soybean plants were randomly selected from the central rows to determine yield components and to avoid border effects. The number of pods per plant (NPP) was counted as the total number of mature pods on each sampled plant. After threshing the sampled plants, the number of grains per plant (NGP) was determined by counting the total number of filled grains obtained from each plant. The 100-grain weight, expressed as hundred-grain mass (HGM), was measured by randomly selecting 100 filled grains from each plot after natural drying and weighing them using an electronic balance. For grain yield determination, border rows were removed to minimize edge effects, and soybean plants from the effective harvest area of each plot were harvested at maturity. The harvested plants were naturally dried, threshed, and weighed. Grain yield was then converted to yield per unit area and expressed as kg ha^-^¹.

Equation 4 calculates nitrogen fertilizer use efficiency of soybean:

(4) $$NPE=\frac{Y}{N}$$

where *NPE* is the nitrogen fertilizer use efficiency, kg kg^-1^; *Y* is the yield; and *N* is the nitrogen applied, kg ha^-1^.

### 2.4 Statistical analysis of data

The data from the experiment was processed using WPS 2022. For graphing, Origin 22 software was used while SPSS 27.0 was used for statistical analyses. Analysis of variance (ANOVA) was performed using R version 3.6.3 (R Development Core Team, 2012) at 0.05 level of significance. Additionally, correlation analysis was performed using Pearson’s correlation coefficient.

## 3 Results

### 3.1 Soil hydrothermal environment

#### 3.1.1 Soil moisture content

**Figure 2:**
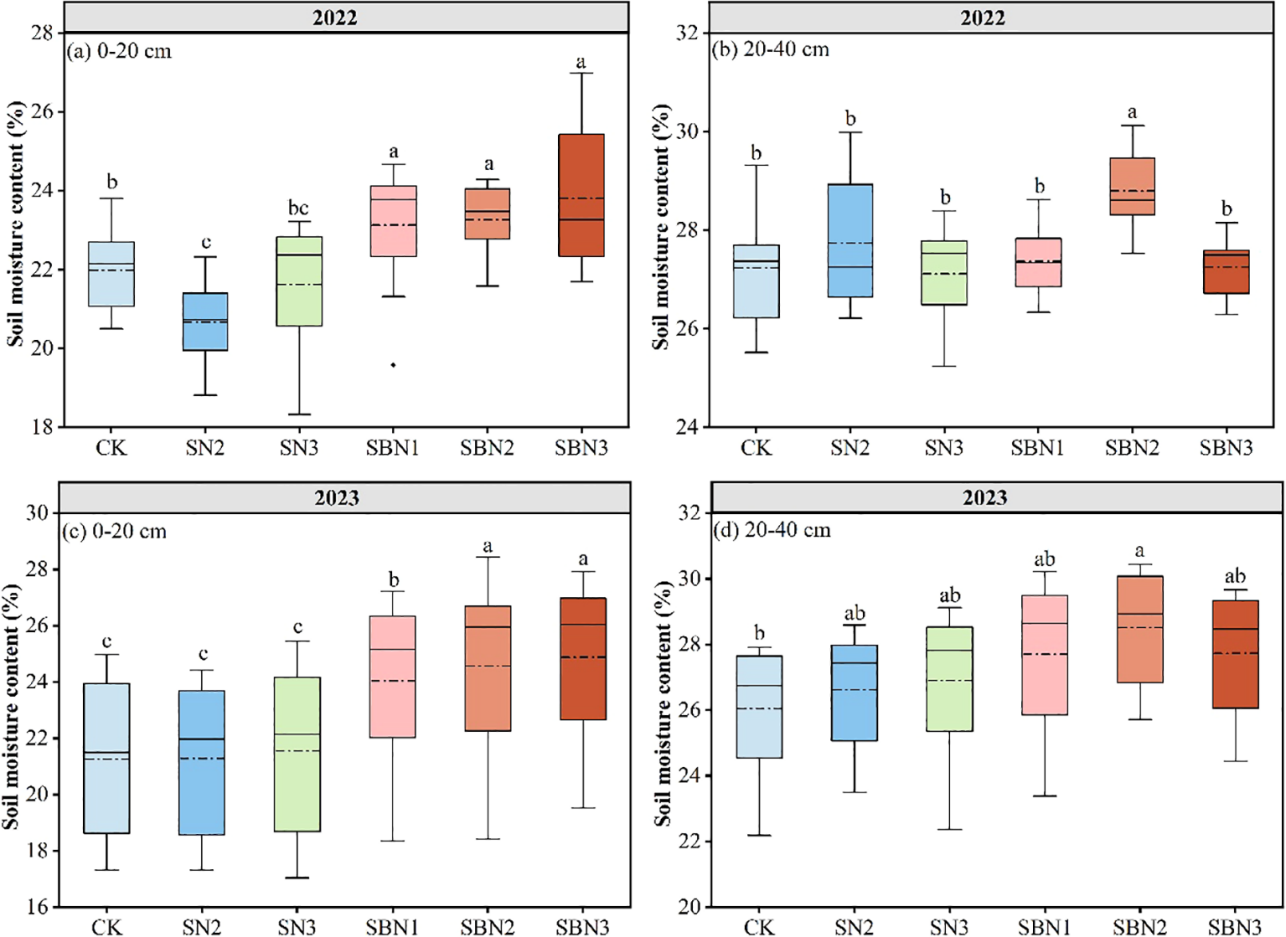
Effects of straw and biochar treatments on growing-season average soil water content under different nitrogen application rates. CK (conventional N with full straw return), SN2 (80% N with full straw), SN3 (60% N with full straw), SBN1 (conventional N with straw - biochar mix), SBN2 (80% N with straw - biochar mix), and SBN3 (60% N with straw - biochar mix). Different lowercase letters indicate significant differences among treatments (*P* < 0.05).

The SBN combination significantly (*P<*0.05) increased the soil moisture content (SMC) in the 0–20 cm layer compared to the SN combination (**Figure 2**). In the 20–40 cm soil layer, differences in SMC among treatments were generally smaller than those in the 0–20 cm layer; however, SBN2 still showed significantly higher SMC than CK in both years (*P* < 0.05). Specifically, the average SMC in the 0–20 cm soil layer for the SBN combination was 9.0% in 2022 and 14.3% higher in 2023 compared to the SN combination. Furthermore, different rates of nitrogen application did not significantly affect the SMC in either the 0–20 cm or 20–40 cm layers.

#### 3.1.2 Soil temperature and soil accumulation temperature

**Figure 3:**
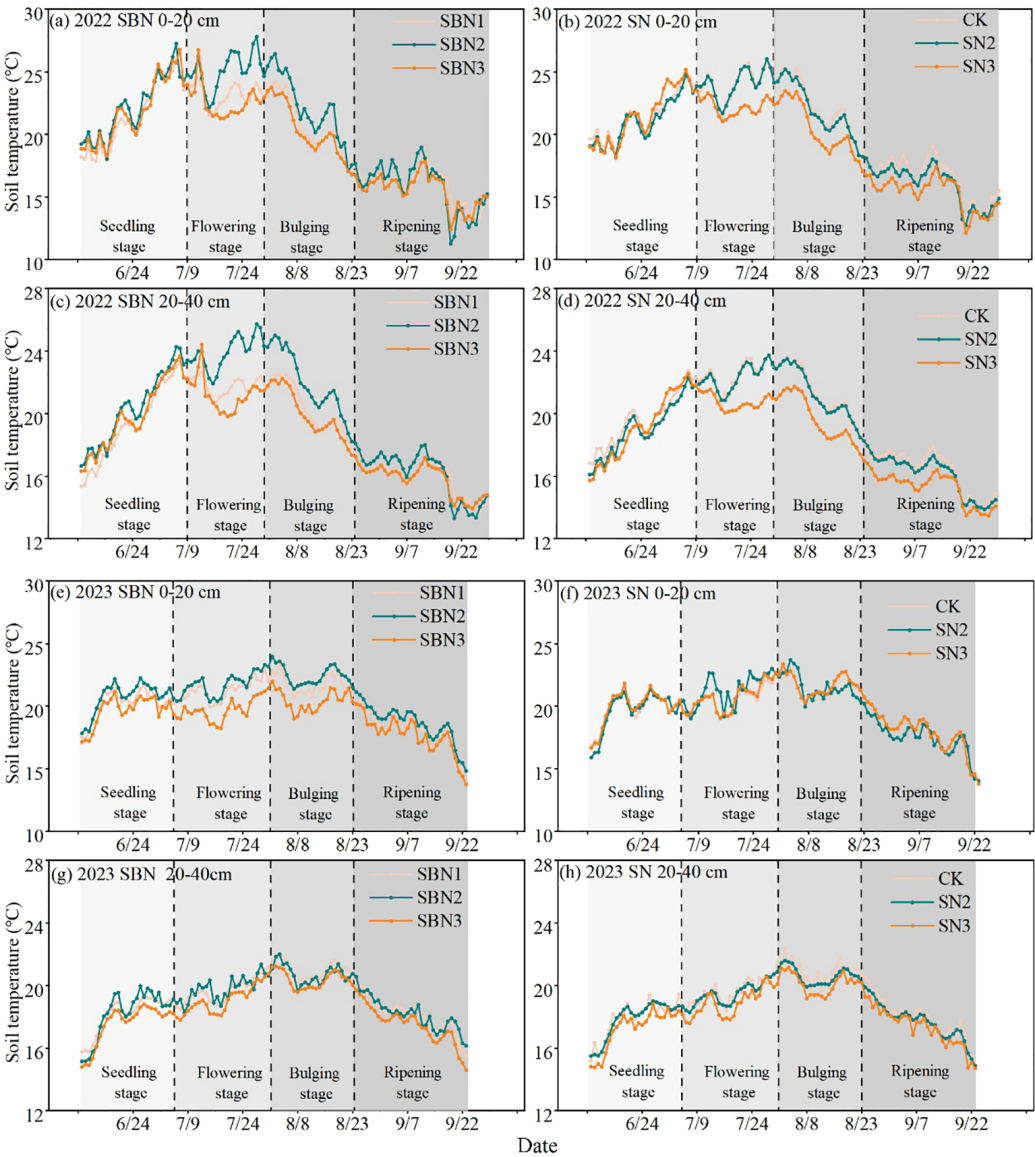
Effects of straw and biochar treatments on soil temperature under different nitrogen applications. CK (conventional N with full straw return), SN2 (80% N with full straw), SN3 (60% N with full straw), SBN1 (conventional N with straw - biochar mix), SBN2 (80% N with straw - biochar mix), and SBN3 (60% N with straw - biochar mix). **(a)** SBN treatments in the 0–20 cm soil layer in 2022; **(b)** SN treatments in the 0–20 cm soil layer in 2022; **(c)** SBN treatments in the 20–40 cm soil layer in 2022; **(d)** SN treatments in the 20–40 cm soil layer in 2022; **(e)** SBN treatments in the 0–20 cm soil layer in 2023; **(f)** SN treatments in the 0–20 cm soil layer in 2023; **(g)** SBN treatments in the 20–40 cm soil layer in 2023; **(h)** SN treatments in the 20–40 cm soil layer in 2023.

Soil temperature showed similar seasonal dynamics across treatments during the two-year experiment (**Figure 3**). In both 2022 and 2023, soil temperature increased gradually from the seedling stage to the flowering stage, reached a peak around mid-to-late July, and then declined during the later growth stages. This pattern was consistent across both SN and SBN combinations.

Compared with the SN combination, the SBN combination slightly increased soil temperature at both 0–20 cm and 20–40 cm depths. Across the two years, the mean daily soil temperature under SBN was approximately 0.1 °C higher in the 0–20 cm layer and 0.3 °C higher in the 20–40 cm layer than that under SN. Although the magnitude of this increase was small, it indicates that straw–biochar co-application tended to maintain a slightly warmer soil environment during the soybean growing season.

Accumulated soil temperature differed more clearly among treatments (**Table 5**). Compared with SN2, SBN2 increased accumulated soil temperature by 2.4% and 4.2% in the 0–20 cm and 20–40 cm layers, respectively, in 2022. Among the SBN treatments, SBN2 generally showed the highest accumulated soil temperature, followed by SBN1, whereas SBN3 showed a lower value. This indicates that straw–biochar co-application combined with 80% N application created a more favorable soil thermal condition than either full straw return alone or 60% N application.

**Table 5:** Effects of mixed application of biochar and straw on cumulative temperature of tillage soil under different nitrogen.

Treatment	2022		2023	
	0–20 cm	20–40 cm	0–20 cm	20–40 cm
CK	2215.2 bc	2120.4 b	2100.1 c	2008.6 b
SN2	2258.7 b	2149.6 b	2100.2 c	1994.0 c
SN3	2167.5 c	2050.2 c	2041.9 d	1940.8 c
SBN1	2290.1 a	2179.6 b	2138.3 b	2029.5 a
SBN2	2312.0 a	2238.9 a	2199.2 a	2029.7 a
SBN3	2192.1 c	2104.7 c	2109.8 c	1962.5 c

CK (conventional N with full straw return), SN2 (80% N with full straw), SN3 (60% N with full straw), SBN1 (conventional N with straw - biochar mix), SBN2 (80% N with straw - biochar mix), and SBN3 (60% N with straw - biochar mix). Values followed by different lowercase letters within the same column indicate significant differences among treatments at *P* < 0.05.

### 3.2 Effects of different treatments on soil nutrients

#### 3.2.1 Changes in available soil nutrients

**Figure 4:**
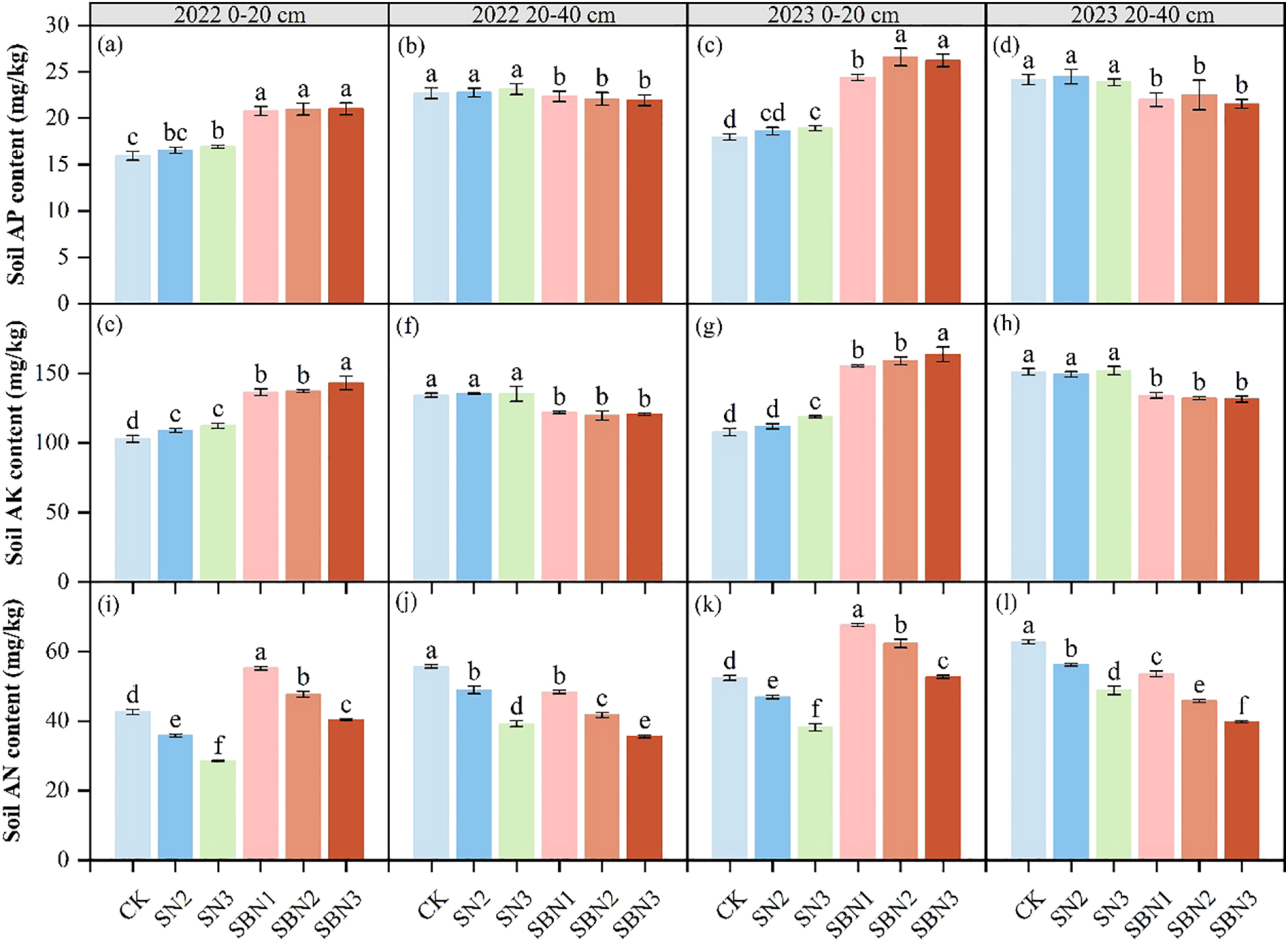
Effect of straw and biochar treatments on available soil nutrients (available phosphorus (AP); available potassium (AK); and available nitrogen (AN)) under different nitrogen application rates. CK (conventional N with full straw return), SN2 (80% N with full straw), SN3 (60% N with full straw), SBN1 (conventional N with straw - biochar mix), SBN2 (80% N with straw - biochar mix), and SBN3 (60% N with straw - biochar mix). Different lowercase letters indicate significant differences among treatments within the same year and soil layer at *P* < 0.05. **(a)** AP content in the 0–20 cm soil layer in 2022; **(b)** AP content in the 20–40 cm soil layer in 2022; **(c)** AP content in the 0–20 cm soil layer in 2023; **(d)** AP content in the 20–40 cm soil layer in 2023; **(e)** AK content in the 0–20 cm soil layer in 2022; **(f)** AK content in the 20–40 cm soil layer in 2022; **(g)** AK content in the 0–20 cm soil layer in 2023; **(h)** AK content in the 20–40 cm soil layer in 2023; **(i)** AN content in the 0–20 cm soil layer in 2022; **(j)** AN content in the 20–40 cm soil layer in 2022; **(k)** AN content in the 0–20 cm soil layer in 2023; **(l)** AN content in the 20–40 cm soil layer in 2023.

Soil available nutrients were analyzed by comparing biomass-return methods first and then N application rates within each biomass-return method (**Figure 4**). Overall, straw–biochar co-application significantly improved soil available nutrient status, especially in the 0–20 cm soil layer.

Compared with the SN combination, the SBN combination increased available nitrogen (AN), available phosphorus (AP), and available potassium (AK) in the 0–20 cm layer. In 2022, AN, AP, and AK under SBN increased by 48.9%, 56.8%, and 28.4%, respectively, compared with SN. In 2023, the corresponding increases were 47.5%, 71.7%, and 41.2%, respectively. These results indicate that the nutrient-improving effect of straw–biochar co-application became more evident in the second year, particularly for AP and AK.

The response of available nutrients to N application rate differed among nutrient types. AN generally increased with increasing N application rate under both SN and SBN combinations, indicating that soil available N was directly affected by fertilizer N input. In contrast, AP and AK showed weaker responses to N application rate, suggesting that their variation was more closely related to the biomass-return method than to N input. Among the SBN treatments, SBN2 maintained relatively high AN, AP, and AK contents while reducing N input by 20%, indicating that straw–biochar co-application improved nutrient availability under moderate N reduction.

In the 20–40 cm soil layer, changes in available nutrients were less pronounced than those in the 0–20 cm layer. This suggests that the positive effect of straw–biochar co-application on available nutrient supply was mainly concentrated in the topsoil, where biochar was incorporated.

#### 3.2.2 Changes in total soil nutrients and soil organic carbon

**Figure 5:**
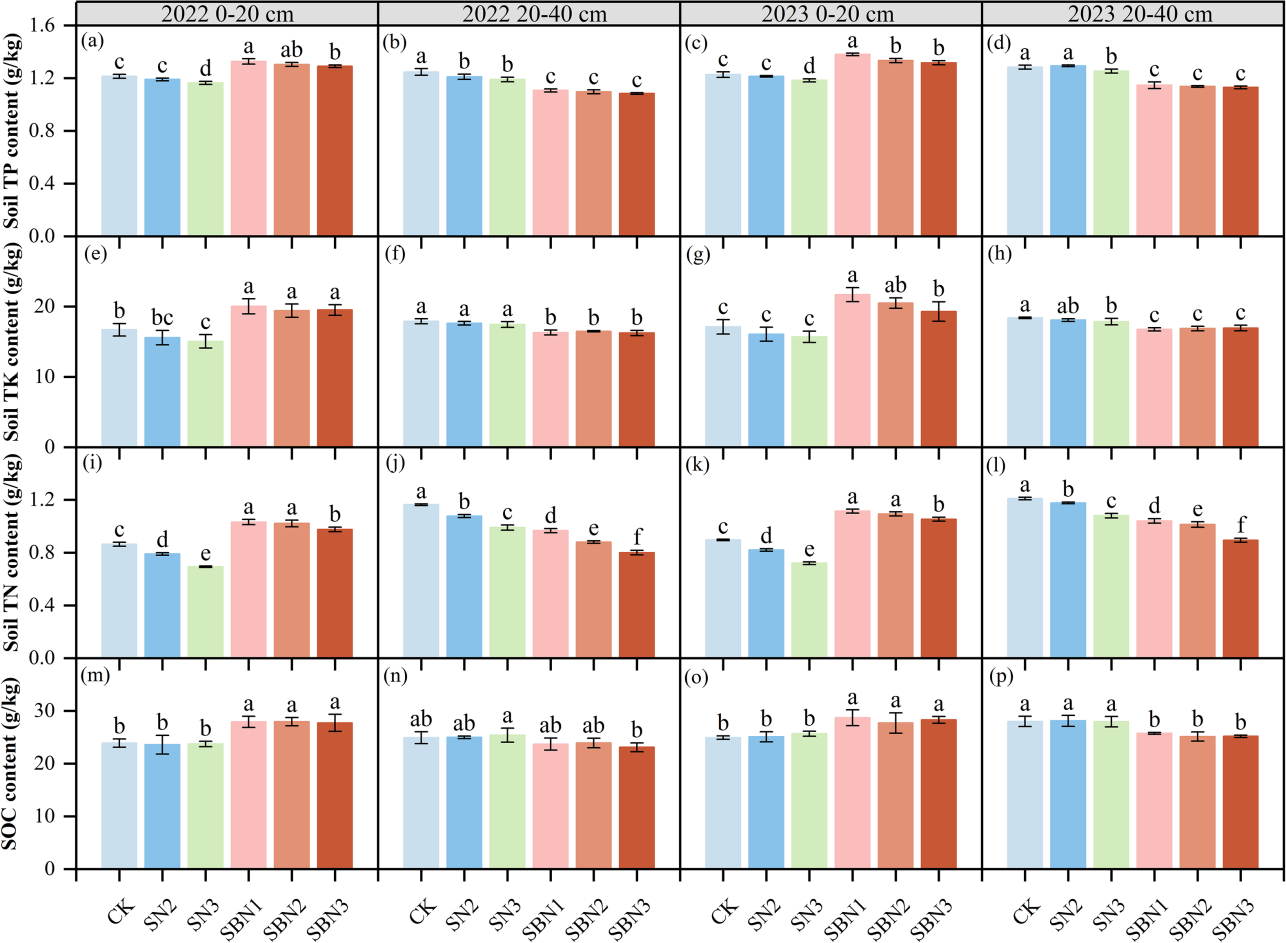
Effects of straw and biochar treatments on total soil nutrients (total phosphorus (TP); total potassium (TK); total nitrogen (TN); soil organic carbon (SOC)) under different nitrogen application rates. CK (conventional N with full straw return), SN2 (80% N with full straw), SN3 (60% N with full straw), SBN1 (conventional N with straw - biochar mix), SBN2 (80% N with straw - biochar mix), and SBN3 (60% N with straw - biochar mix). Different lowercase letters indicate significant differences among treatments within the same year and soil layer at *P* < 0.05. #**(a)** TP content in the 0–20 cm soil layer in 2022; **(b)** TP content in the 20–40 cm soil layer in 2022; **(c)** TP content in the 0–20 cm soil layer in 2023; **(d)** TP content in the 20–40 cm soil layer in 2023; **(e)** TK content in the 0–20 cm soil layer in 2022; **(f)** TK content in the 20–40 cm soil layer in 2022; **(g)** TK content in the 0–20 cm soil layer in 2023; **(h)** TK content in the 20–40 cm soil layer in 2023; **(i)** TN content in the 0–20 cm soil layer in 2022; **(j)** TN content in the 20–40 cm soil layer in 2022; **(k)** TN content in the 0–20 cm soil layer in 2023; **(l)** TN content in the 20–40 cm soil layer in 2023; **(m)** SOC content in the 0–20 cm soil layer in 2022; **(n)** SOC content in the 20–40 cm soil layer in 2022; **(o)** SOC content in the 0–20 cm soil layer in 2023; **(p)** SOC content in the 20–40 cm soil layer in 2023.

The contents of total nitrogen (TN), total phosphorus (TP), total potassium (TK), and soil organic carbon (SOC) were also affected by biomass-return method and soil depth (**Figure 5**). Compared with the SN combination, the SBN combination significantly increased total nutrient contents and SOC in the 0–20 cm soil layer.

In 2022, the SBN combination increased TN, TP, TK, and SOC by 61.4%, 9.9%, 23.2%, and 24.2%, respectively, compared with the SN combination. In 2023, these increases reached 67.3%, 11.2%, 25.7%, and 33.2%, respectively. These results show that straw–biochar co-application was more effective than straw return alone in improving topsoil nutrient accumulation and SOC storage.

Within each biomass-return method, TN generally responded more strongly to N application rate than TP, TK, and SOC. TN tended to be higher under N1 and N2 than under N3, indicating that excessive N reduction weakened soil N accumulation. However, TP, TK, and SOC were less sensitive to N application rate and were more strongly affected by the addition of biochar. Among the SBN treatments, SBN2 maintained high TN and SOC levels, suggesting that 80% N application combined with straw–biochar co-application could sustain soil fertility while reducing N fertilizer input.Similar to available nutrients, the improvement in total nutrients and SOC was mainly observed in the 0–20 cm soil layer, while the changes in the 20–40 cm layer were relatively limited.

### 3.3 Effects of different treatments on the growth of soybean plants

#### 3.3.1 Plant height, stem thickness and leaf area

**Figure 6:**
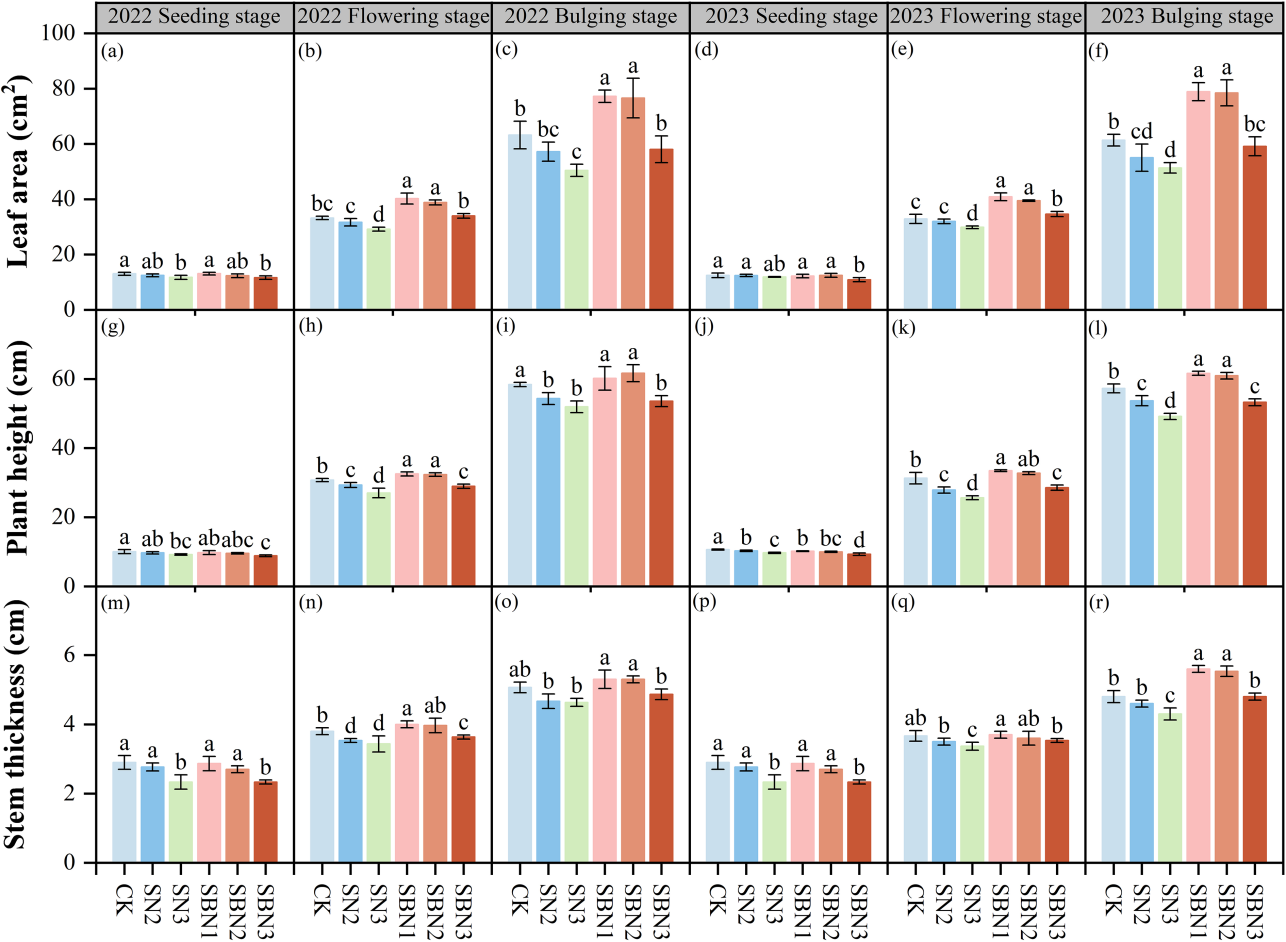
Effect of straw and biochar treatments on plant height, stem thickness and leaf area of soybean under different nitrogen application rates. CK (conventional N with full straw return), SN2 (80% N with full straw), SN3 (60% N with full straw), SBN1 (conventional N with straw - biochar mix), SBN2 (80% N with straw - biochar mix), and SBN3 (60% N with straw - biochar mix). Different lowercase letters indicate significant differences among treatments within the same year and growth stage at *P* < 0.05. #**(a)** Leaf area at the seedling stage in 2022; **(b)** Leaf area at the flowering stage in 2022; **(c)** Leaf area at the bulging stage in 2022; **(d)** Leaf area at the seedling stage in 2023; **(e)** Leaf area at the flowering stage in 2023; **(f)** Leaf area at the bulging stage in 2023; **(g)** Plant height at the seedling stage in 2022; **(h)** Plant height at the flowering stage in 2022; **(i)** Plant height at the bulging stage in 2022; **(j)** Plant height at the seedling stage in 2023; **(k)** Plant height at the flowering stage in 2023; **(l)** Plant height at the bulging stage in 2023; **(m)** Stem thickness at the seedling stage in 2022; **(n)** Stem thickness at the flowering stage in 2022; **(o)** Stem thickness at the bulging stage in 2022; **(p)** Stem thickness at the seedling stage in 2023; **(q)** Stem thickness at the flowering stage in 2023; **(r)** Stem thickness at the bulging stage in 2023.

Soybean growth traits were compared between the SN and SBN combinations and among N application rates within each biomass-return method (**Figure 6**). In general, plant height, stem diameter, and leaf area were more strongly improved by straw–biochar co-application during the middle and later growth stages than at the seedling stage.

At the seedling stage, differences in plant growth traits between SN and SBN were relatively small, indicating that the early growth response to straw–biochar co-application was limited. However, at the flowering and podding stages, the SBN combination significantly increased plant height, stem diameter, and leaf area compared with the SN combination. Across the two years, straw–biochar co-application increased leaf area by approximately 23.6%, plant height by 8.9%, and stem diameter by 8.6% compared with straw return alone.

Within the SN combination, plant height, stem diameter, and leaf area generally decreased as N application rate declined, showing that soybean growth was sensitive to N reduction under straw return alone. In contrast, under the SBN combination, SBN2 maintained growth traits close to those of SBN1, whereas SBN3 showed lower values. These results indicate that straw–biochar co-application could partly compensate for a 20% reduction in N input, but not for a 40% reduction.

#### 3.3.2 Pn and Gs

**Figure 7:**
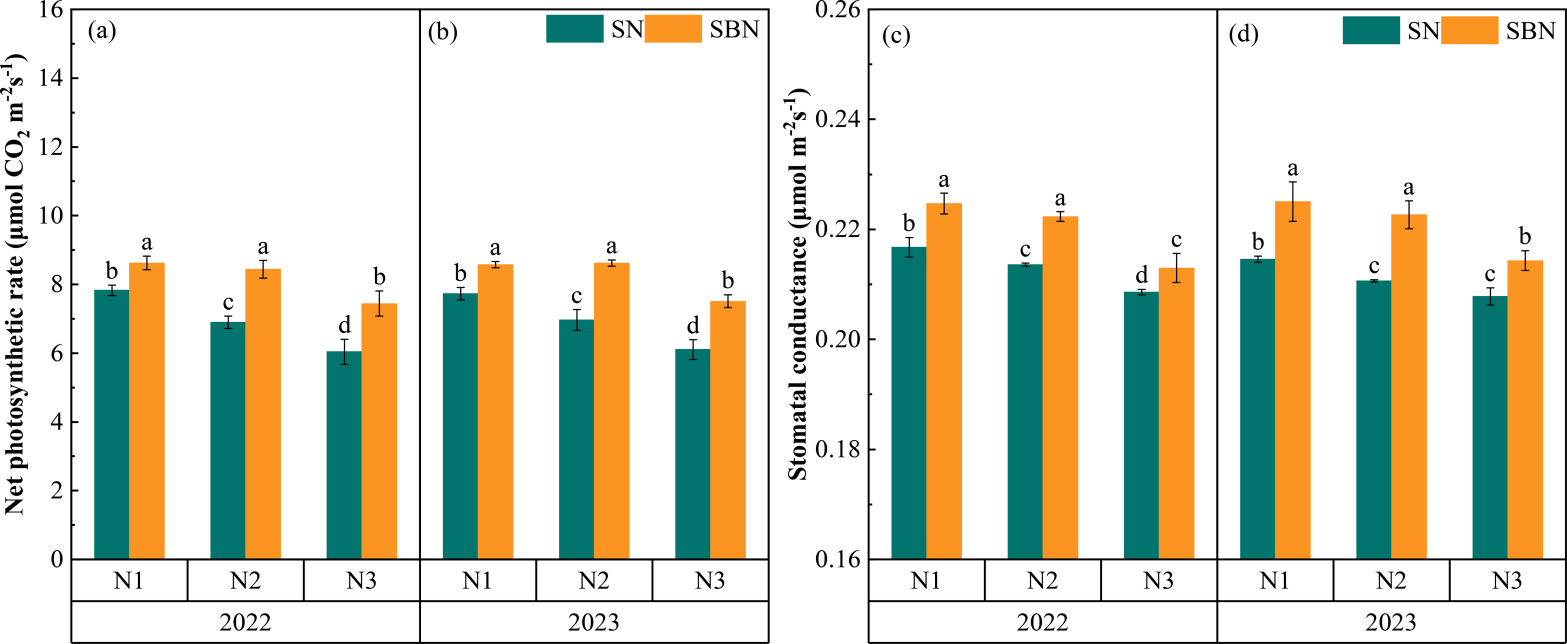
Effects of straw and biochar treatments on soybean photosynthesis under different nitrogen application rates. CK (conventional N with full straw return), SN2 (80% N with full straw), SN3 (60% N with full straw), SBN1 (conventional N with straw - biochar mix), SBN2 (80% N with straw - biochar mix), and SBN3 (60% N with straw - biochar mix). Different lowercase letters indicate significant differences among treatments within the same year at *P* < 0.05. **(a)** Net photosynthetic rate (Pn) in 2022; **(b)** Net photosynthetic rate (Pn) in 2023; **(c)** Stomatal conductance (Gs) in 2022; **(d)** Stomatal conductance (Gs) in 2023.

The photosynthetic parameters, net photosynthetic rate (Pn) and stomatal conductance (Gs) were measured at the flowering stage in soybean plants (**Figure 7**). Compared with the SN combination, the SBN combination increased both Pn and Gs under all N application rates in both years. Across the two years, straw–biochar co-application increased Pn and Gs by an average of 20.6% and 3.1%, respectively, compared with full straw return alone.

Under the SN combination, Pn decreased with decreasing N application rate. For example, Pn was approximately 7.7–7.8 μmol CO_2_ m^-^² s^-^¹ under N1, declined to about 6.8–7.0 μmol CO_2_ m^-^² s^-^¹ under N2, and further declined to about 6.0 μmol CO_2_ m^-^² s^-^¹ under N3. Under the SBN combination, Pn remained higher across all N levels, with SBN1 and SBN2 showing comparable values of approximately 8.4–8.7 μmol CO_2_ m^-^² s^-^¹, while SBN3 decreased to about 7.3–7.5 μmol CO_2_ m^-^² s^-^¹.

A similar pattern was observed for Gs. SBN treatments generally showed higher Gs than the corresponding SN treatments. Among the SBN treatments, SBN1 and SBN2 maintained higher Gs, whereas SBN3 showed a decline. These results indicate that straw–biochar co-application improved soybean photosynthetic capacity and stomatal activity, and that SBN2 maintained physiological performance comparable to SBN1 despite a 20% reduction in N input.

#### 3.3.3 SPAD values

**Figure 8:**
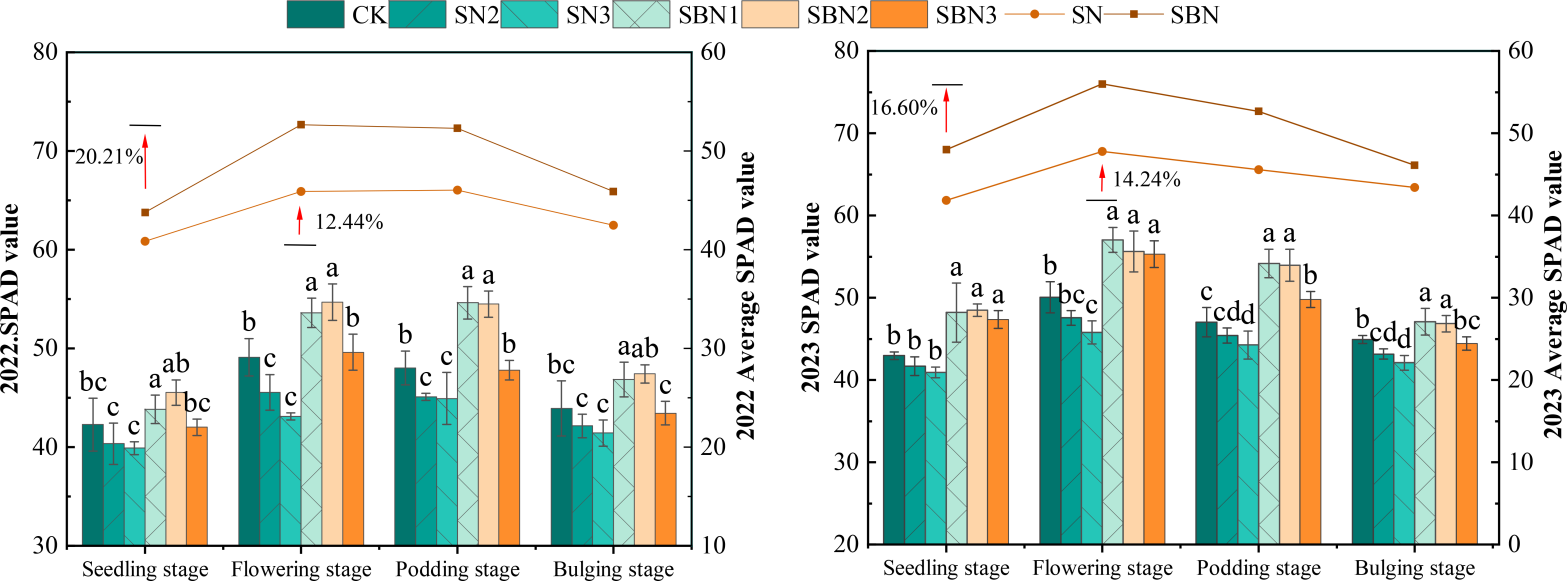
Effect of straw and biochar treatments on chlorophyll values of soybean under different nitrogen applications. CK (conventional N with full straw return), SN2 (80% N with full straw), SN3 (60% N with full straw), SBN1 (conventional N with straw - biochar mix), SBN2 (80% N with straw - biochar mix), and SBN3 (60% N with straw - biochar mix). Different lowercase letters indicate significant differences among treatments within the same year and growth stage at *P* < 0.05.

SPAD values varied across growth stages, years, biomass-return methods, and N application rates (**Figure 8**). Across all treatments, SPAD values generally increased from the seedling stage to the flowering stage, remained relatively high during the podding period, and decreased during the bulging stage. This pattern indicates that leaf chlorophyll status peaked during the flowering and podding stages.

Compared with the SN combination, the SBN combination consistently increased SPAD values in both 2022 and 2023. In 2022, the average SPAD value under the SBN combination was 11.0% higher than that under the SN combination. In 2023, the increase reached 13.5%. The line plots also showed that the average SPAD values of the SBN combination were higher than those of the SN combination across all growth stages, indicating a consistent improvement in leaf chlorophyll status under straw–biochar co-application.

Within the SN combination, SPAD values decreased with decreasing N application rate, especially at the flowering and podding stages. Under the SBN combination, SBN1 and SBN2 generally showed similar SPAD values, while SBN3 was lower. For example, at the flowering stage, SBN1 and SBN2 maintained relatively high SPAD values of approximately 55–57, whereas SBN3 declined to about 50–55 depending on the year. This indicates that 80% N application under straw–biochar co-application was sufficient to maintain leaf chlorophyll content, while 60% N application caused a clear decline in SPAD values.

#### 3.4 Soybean yield components

Soybean yield components and grain yield were analyzed by comparing individual treatments, with particular attention to whether SBN2 could maintain yield under a 20% reduction in N input (**Table 6**). The number of pods per plant (NPP), number of grains per plant (NGP), and hundred-grain mass (HGM) showed trends generally consistent with grain yield.

**Table 6:** Effects of straw and biochar treatments on soybean yield, yield components, NPE and WUE_n_ at different nitrogen application rates.

Year	Treatment	WUE_n_	NPP	NGP	HGM	Yield	NPE
2022	CK	32.7 b	24.3 a	52.0 a	22.1 c	2494.4 ab	41.5 d
	SN2	31.7 c	23.3 a	50.4 b	20.4 d	2325.8 b	48.6 c
	SN3	29.9 c	20.6 b	46.2 c	21.6 c	2019.4 c	56.9 b
	SBN1	38.2 a	28.3 b	51.6 b	24.7 b	2593.7 a	43.3 cd
	SBN2	37.4 a	33.3 a	59.3 a	28.1 a	2581.6 a	60.3 a
	SBN3	34.9 b	22.0 c	43.3 c	20.1 d	2233.2 bc	62.3 a
2023	CK	32.9 bc	24.6 bc	51.7 b	24.5 c	2413.4 ab	40.2 c
	SN2	32.5 c	23.0 c	50.2 b	22.4 d	2347.1 b	48.9 b
	SN3	31.2 c	20.3 d	40.4 c	22.2 d	2147.2 c	59.4 a
	SBN1	38.1 a	27.3 b	50.6 b	25.3 b	2510.1 a	41.3 c
	SBN2	37.9 a	32.0 a	57.4 a	27.5 a	2564.5 a	58.6 a
	SBN3	36.9 b	21.6 d	41.4 c	23.1 d	2199.4 c	61.1 a

CK (conventional N with full straw return), SN2 (80% N with full straw), SN3 (60% N with full straw), SBN1 (conventional N with straw - biochar mix), SBN2 (80% N with straw - biochar mix), and SBN3 (60% N with straw - biochar mix). WUE_n_, NPP, NGP, HGM, and NPE represent leaf intrinsic water use efficiency, number of pods per plant, number of grains per plant, hundred-grain mass, and nitrogen fertilizer productivity, respectively. Values followed by different lowercase letters within the same column indicate significant differences among treatments *P* < 0.05.

Under the SN combination, soybean yield decreased with decreasing N application rate. In 2022, yield decreased from 2494.4 kg ha^-^¹ under CK to 2325.8 kg ha^-^¹ under SN2 and 2019.4 kg ha^-^¹ under SN3. In 2023, yield decreased from 2413.4 kg ha^-^¹ under CK to 2347.1 kg ha^-^¹ under SN2 and 2147.2 kg ha^-^¹ under SN3. These results indicate that reducing N input under full straw return alone reduced soybean yield.

In contrast, under the SBN combination, SBN1 and SBN2 showed no significant difference in soybean yield in either year. In 2022, soybean yield was 2593.7 kg ha^-^¹ under SBN1 and 2581.6 kg ha^-^¹ under SBN2. In 2023, yield was 2510.1 kg ha^-^¹ under SBN1 and 2564.5 kg ha^-^¹ under SBN2. These results indicate that a 20% reduction in N input did not reduce soybean yield when straw and biochar were co-applied.

However, SBN3 produced lower yield than SBN1 and SBN2. The yield under SBN3 was 2233.2 kg ha^-^¹ in 2022 and 2199.4 kg ha^-^¹ in 2023. This suggests that a 40% reduction in N input exceeded the compensatory capacity of straw–biochar co-application. Therefore, SBN2 achieved the best balance between maintaining soybean yield and reducing N fertilizer input.

Yield components further supported this result. In 2022, SBN2 showed the highest NPP, NGP, and HGM among all treatments, with values of 33.3 pods plant^-^¹, 59.3 grains plant^-^¹, and 28.1 g, respectively. In 2023, SBN2 also maintained high values of NPP, NGP, and HGM, reaching 32.0 pods plant^-^¹, 57.4 grains plant^-^¹, and 27.5 g, respectively. These improvements in yield components contributed to the stable soybean yield under SBN2.

Nitrogen fertilizer productivity (NPE) increased as N input decreased. Although SBN3 had relatively high NPE due to the lower N application rate, its yield was significantly reduced. Therefore, NPE alone was not sufficient to identify the optimal treatment. Considering soybean yield, yield components, WUEn, and NPE together, SBN2 was the most appropriate treatment.

### 3.5 Relationships of yield with soil hydrothermal properties, soil nutrient status and plant photosynthetic characteristics

**Figure 9:**
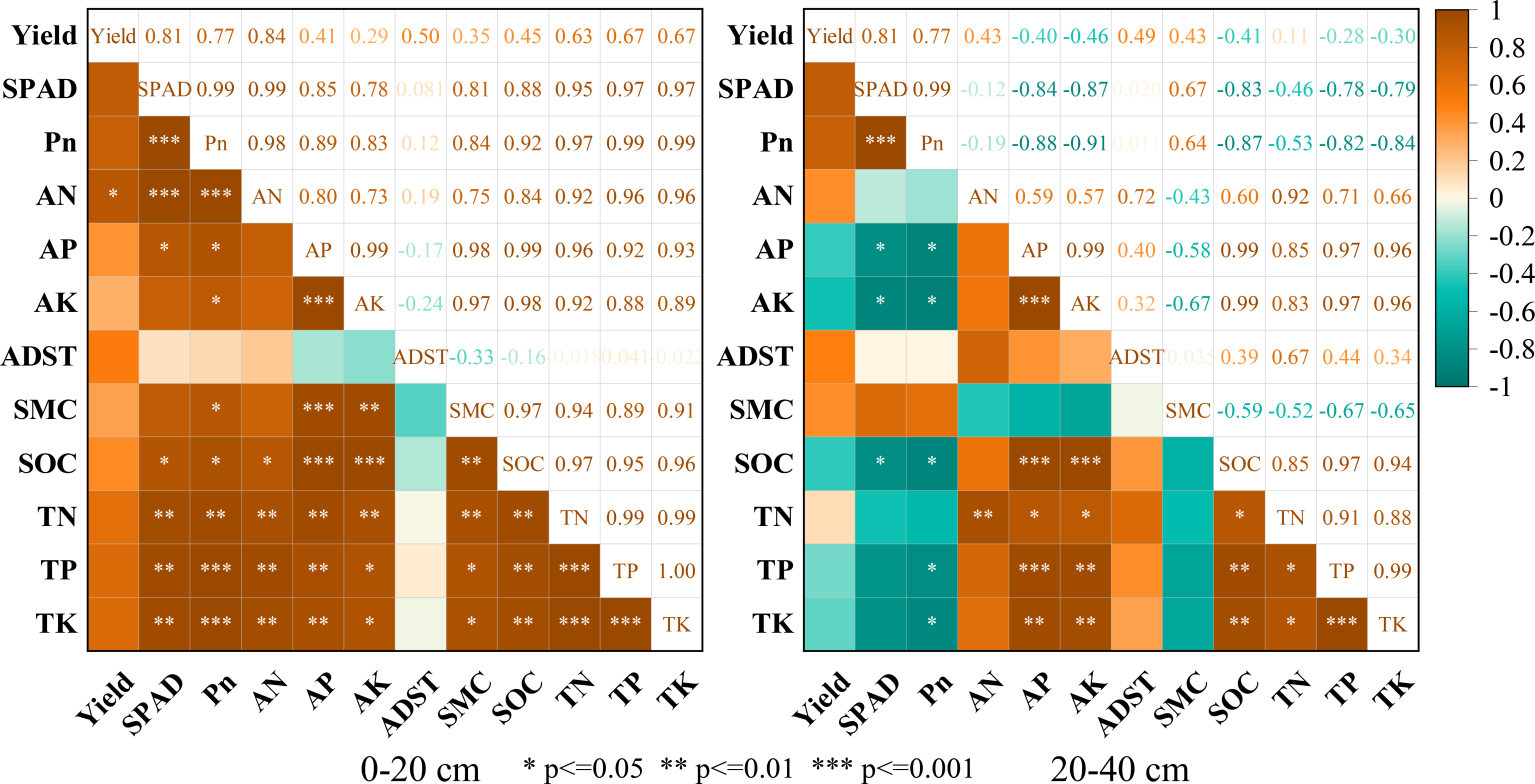
Heat map of correlations between different crop indicators and soil nutrient indicators. The heat map shows Pearson correlation coefficients among soil hydrothermal properties, soil nutrient indicators, soybean physiological traits, and yield. The color scale represents the strength and direction of the correlation. *p<=0.05; **<=0.01; ***p<=0.001.

Soil nutrients and plant growth data at podding and bulking stages and yield during the two-year trial period were selected to assess the relationship between the indicators by Pearson correlation analysis (**Figure 9**). The results demonstrated that all soil nutrient indicators in the 0–20 cm soil layer were positively correlated with soybean yield. Specifically, soil TN, TP, TK and AN were significantly positively correlated with soybean photosynthesis. Conversely, the average daily soil temperature (ADST) exhibited a negative correlation with SMC, AP, AK and SOC. In the 20–40 cm soil layer, soil total and available nutrient contents were not strongly associated with soybean growth or yield. Nevertheless, a significant positive correlation was detected between soil available nutrients and total nutrients in this layer. Additionally, SOC content was closely associated with soil nutrient levels, highlighting its role in nutrient cycling and soil fertility.

## 4 Discussion

### 4.1 Effects of combined application of straw and biochar on the soil hydrothermal environment

Soil moisture and temperature are key environmental factors controlling crop growth, nutrient cycling, and yield formation (Obia et al., 2020). In this study, straw–biochar co-application significantly increased soil moisture content in the 0–20 cm soil layer compared with straw return alone, indicating that biochar played an important role in improving topsoil water retention. This improvement may be attributed to the porous structure, high specific surface area, and hydrophilic functional groups of biochar, which can enhance soil water adsorption and storage capacity (Sadaf et al., 2017; Deng et al., 2024). In addition, straw–biochar co-application may improve soil aggregation and pore structure, thereby enhancing water retention in the plow layer (Wang et al., 2023; Geng et al., 2024).

The effect of straw–biochar co-application on SMC was stronger in the 0–20 cm layer than in the 20–40 cm layer. This depth-dependent response was likely related to the placement of biochar in the upper soil layer (Blanco-Canqui et al., 2024). Because biochar was incorporated into the 0–20 cm soil layer, its effects on soil porosity, water adsorption, and nutrient retention were concentrated mainly in the topsoil (Li et al., 2023). Although differences in the 20–40 cm layer were weaker, SBN2 still maintained relatively high SMC, indicating that the integrated straw–biochar system may also contribute to water redistribution within the 0–40 cm soil profile.

Straw–biochar co-application also affected soil temperature and accumulated soil temperature. In this study, the SBN combination significantly increased mean daily soil temperature and accumulated soil temperature compared with the SN combination, especially under SBN2 (**Figure 3**). Biochar can influence soil thermal conditions by changing soil color, albedo, heat capacity, and thermal conductivity (Xiu et al., 2021; Khaledi et al., 2023). The darker color of biochar-amended soil can increase heat absorption, while changes in soil porosity and water content can further alter soil heat transfer and storage (Wang et al., 2022). However, the effects of biochar on soil temperature are not always consistent. Some studies reported warming effects, whereas others found that biochar could reduce or buffer soil temperature depending on biochar type, soil texture, soil moisture, application rate, and observation period (Obia et al., 2015). Therefore, based on our results and previous studies, straw–biochar co-application should be interpreted as a practice that may buffer soil temperature fluctuations and maintain a more favorable soil thermal environment for soybean growth, rather than simply increasing soil temperature under all conditions.

### 4.2 Effects of combined application of straw and biochar on the soil nutrient environment

Improved soil nutrient status is a key pathway through which straw and biochar affect crop growth and yield (Bass et al., 2016). In this study, straw–biochar co-application significantly increased soil available nutrients, total nutrients, and SOC in the 0–20 cm soil layer compared with straw return alone. This indicates that the combined application of straw and biochar was more effective than straw return alone in improving topsoil fertility. Similar findings have been reported in previous studies, where biochar application increased soil organic carbon, nutrient retention, and nutrient availability, thereby improving soil fertility and crop productivity (Nobile et al., 2022).

The stronger nutrient response in the 0–20 cm layer can be explained by the incorporation depth and nutrient-retention capacity of biochar. Biochar contains mineral nutrients and stable carbon, and its porous structure and surface functional groups can adsorb and retain nutrients such as NH_4_^+^-N, NO_3_^-^-N, K^+^, and phosphate, thereby reducing nutrient leaching and improving nutrient availability during crop growth (Thi et al., 2017; Gou et al., 2022). Ma et al. (2020) also showed that biochar application enhanced ^15^N retention in the soil and improved N utilization by crops, supporting the idea that biochar can partly offset reduced N fertilizer input by improving soil N retention. In the present study, SBN2 maintained relatively high TN and AN under a 20% reduction in N input, which may be related to this nutrient-retention effect.

Straw decomposition is another important source of nutrient release. However, the nutrient contribution of straw depends strongly on decomposition rate, soil temperature, soil moisture, microbial activity, and C/N ratio (Zhao et al., 2019; Wang et al., 2024). Under straw return alone, the release of nutrients from straw may be delayed, especially in the cool black soil region of Northeast China. In the early decomposition stage, soil microorganisms may immobilize available N because of the high C/N ratio of straw, resulting in temporary N competition between microorganisms and crops (Ouyang et al., 2016). This may explain why straw return alone showed weaker improvement in available nutrients and why yield declined more clearly under reduced N input in the SN treatments.

In contrast, straw–biochar co-application may alleviate this limitation. Biochar can improve soil water and temperature conditions, which may promote microbial activity and accelerate straw decomposition (He et al., 2022). At the same time, biochar can adsorb nutrients released during straw decomposition and reduce nutrient leaching losses, thereby acting as a slow-release nutrient reservoir (Rosa et al., 2024). This mechanism is consistent with the higher AN, AP, AK, TN, TK, and SOC observed in the 0–20 cm soil layer under SBN treatments.

SOC accumulation also increased significantly under straw–biochar co-application. This result can be explained by two complementary C inputs: straw provides relatively labile organic carbon, while biochar provides stable aromatic carbon that is resistant to rapid microbial decomposition (Li et al., 2020). Biochar can also promote organic carbon stabilization by enhancing aggregate formation, increasing physical protection of organic matter, and altering microbial carbon use processes (Zhang et al., 2024). Therefore, the combined use of straw and biochar may simultaneously support short-term nutrient cycling and long-term SOC accumulation, which is particularly important for maintaining fertility in degraded black soils.

### 4.3 Effects of combined application of straw and biochar on soybean physiological traits

Soybean photosynthetic performance was improved by straw–biochar co-application, as indicated by higher SPAD values, net photosynthetic rate (Pn), and stomatal conductance (Gs). These improvements were closely associated with the enhanced soil water and nutrient conditions under SBN treatments (Helaoui et al., 2023). Adequate soil moisture helps maintain stomatal opening and CO_2_ assimilation, while sufficient nutrient availability, especially N and K, supports chlorophyll synthesis, enzyme activity, and photosynthetic carbon fixation (Xia et al., 2024). A global synthesis by He et al. (2020) also reported that biochar amendment can improve photosynthesis and biomass accumulation in C_3_ crops, which is consistent with the increase in Pn observed in this study.

The increase in SPAD values under the SBN combination indicates that straw–biochar co-application improved leaf chlorophyll status. Nitrogen is a structural component of chlorophyll and photosynthetic proteins, and N deficiency generally reduces leaf chlorophyll concentration and photosynthetic capacity (Qiang et al., 2023). In this study, the SBN combination increased topsoil AN and TN, which likely provided more available N for chlorophyll synthesis and leaf physiological activity. The strong positive correlation between AN and SPAD in the 0–20 cm soil layer further supports this explanation.

The contrasting relationship between AN and SPAD in different soil layers is particularly important. In the 0–20 cm layer, AN was strongly positively correlated with SPAD, whereas in the 20–40 cm layer, the correlation was weak or negative (**Figure 9**). This suggests that topsoil available N had a more direct effect on leaf chlorophyll formation than available N in the deeper soil layer. This may be because biochar was incorporated into the 0–20 cm layer, where nutrient retention and microbial nutrient transformation were more active (Borchard et al., 2019). In addition, soybean nutrient uptake during key growth stages is closely associated with the plow layer, where root activity and fertilizer-derived nutrient availability are usually higher (Xia et al., 2024). Therefore, nutrient improvements in the 0–20 cm layer may have had a more immediate effect on SPAD, Pn, and yield formation.

Although straw–biochar co-application generally improved SPAD values, SBN3 showed a significant reduction compared with SBN1 and SBN2. This result suggests that a 40% reduction in N input exceeded the compensatory capacity of straw–biochar co-application. Previous studies have shown that insufficient N supply can limit chlorophyll biosynthesis, reduce photosynthetic enzyme activity, and restrict dry matter accumulation (Yang et al., 2023). Therefore, although biochar improved nutrient retention, the N input under SBN3 was likely insufficient to maintain leaf N status and chlorophyll synthesis during key growth stages. This explains the lower SPAD values, Pn, and yield observed under SBN3.

The responses of Pn and Gs further confirmed the physiological advantage of SBN2. SBN2 maintained photosynthetic performance comparable to SBN1 despite a 20% reduction in N input, indicating that straw–biochar co-application improved the efficiency of N use by enhancing soil nutrient retention and water availability. Similar findings have been reported in studies showing that biochar combined with N fertilizer improved photosynthetic performance, nutrient uptake, and crop yield (He et al., 2020; Helaoui et al., 2023). However, the lower Pn and Gs under SBN3 indicate that excessive N reduction limited leaf physiological activity, ultimately contributing to yield decline.

### 4.4 Effects of combined application of straw and biochar on soybean yield

Soybean yield was closely associated with yield components, including the number of pods per plant, number of grains per plant, and hundred-grain mass (Bianchi et al., 2020). In this study, the trends in these yield components were generally consistent with grain yield, indicating that treatment-induced yield changes were caused by combined effects on pod formation, grain number, and grain filling. Similar relationships between photosynthetic performance, nutrient supply, and yield components have been reported in soybean and other grain crops (Yang et al., 2024).

Under straw return alone, soybean yield decreased as N application rate decreased. This suggests that straw return alone could not fully compensate for reduced N fertilizer input. Although straw return can improve soil structure and organic matter over time, its short-term nutrient release may be limited by slow decomposition and microbial N immobilization (Zhao et al., 2019; Wang et al., 2024). Therefore, reduced N input under SN treatments may have limited soil available N during critical growth stages, resulting in weaker crop growth, lower photosynthetic performance, and reduced yield.

In contrast, SBN2 maintained soybean yield at a level comparable to SBN1 despite a 20% reduction in N input. This result indicates that straw–biochar co-application can partially replace chemical N fertilizer input by improving soil water retention, nutrient availability, SOC accumulation, and photosynthetic performance. Previous studies have shown that biochar can improve crop productivity by enhancing soil fertility, reducing nutrient losses, improving N retention, and promoting nutrient use efficiency (Yu et al., 2017). Ali et al. (2020) also reported that biochar combined with N fertilizer improved crop yield and N metabolism, supporting the role of biochar in improving fertilizer efficiency.

However, SBN3 significantly reduced soybean yield compared with SBN1 and SBN2. Although NPE under SBN3 was relatively high because of the lower N input, its grain yield was not maintained. This indicates that NPE alone should not be used as the only criterion for identifying the optimal treatment. A treatment with lower N input may show higher NPE mathematically, but if yield declines significantly, it is not agronomically optimal. Therefore, soybean yield, yield components, physiological performance, soil nutrient status, WUEn, and NPE should be evaluated together.

It is also important to note that the effect of biochar on crop yield is context-dependent. Some studies have reported significant yield increases after biochar application, whereas others found limited or inconsistent effects depending on soil type, climate, crop species, biochar properties, and application rate (Jeffery et al., 2011). In the present study, the positive response under SBN2 suggests that moderate biochar application combined with straw return and reduced N fertilizer is suitable for soybean production in the black soil region. However, long-term field monitoring is still needed to determine whether these benefits persist over multiple cropping cycles.

### 4.5 Relationships between soil properties, physiological traits, and soybean yield

The correlation analysis further clarified the mechanism by which straw–biochar co-application improved soybean yield. In the 0–20 cm soil layer, soil available nutrients and SOC were positively correlated with SPAD, Pn, and yield. This indicates that improved topsoil fertility directly contributed to enhanced leaf physiological activity and yield formation. Similar findings have been reported in previous studies, where biochar increased crop productivity by improving soil nutrient retention, SOC accumulation, soil water status, and nutrient availability (Sadaf et al., 2017; Li et al., 2023; Luo et al., 2024).

The positive correlations among SOC, soil nutrients, SPAD, and yield also suggest that SOC plays an important role in regulating soil fertility and crop productivity. SOC can improve soil structure, enhance nutrient retention, support microbial activity, and increase soil water-holding capacity (Cotrufo et al., 2011; Nobile et al., 2022; Zhang et al., 2024). In this study, SBN increased SOC in the 0–20 cm layer, which may have contributed to improved nutrient supply and better physiological performance. This is consistent with previous studies showing that straw return and biochar application can increase SOC and improve crop growth by affecting aggregate formation and nutrient cycling (Geng et al., 2024; Zhang et al., 2024).

The contrasting correlations between AN and SPAD in the 0–20 cm and 20–40 cm soil layers further highlight the importance of topsoil nutrient supply. AN in the 0–20 cm layer was strongly positively correlated with SPAD, whereas AN in the 20–40 cm layer showed a weak or negative correlation. This suggests that topsoil available N was more important for regulating chlorophyll status than available N in the deeper soil layer. Since the 0–20 cm layer was the main biochar incorporation layer and a key zone for nutrient transformation and root uptake, nutrient improvements in this layer likely had a stronger effect on leaf N status, photosynthesis, and yield (Ma et al., 2020; Li et al., 2023; Xia et al., 2024).

Overall, the results support a soil–plant pathway by which straw–biochar co-application improved soybean yield: it enhanced topsoil water retention and nutrient availability, increased SOC accumulation, improved SPAD and photosynthetic capacity, and finally promoted yield formation. Among all treatments, SBN2 provided the most favorable balance between soil improvement, physiological performance, yield maintenance, and N fertilizer reduction. This finding supports the potential of straw–biochar co-application as a practical strategy for reducing N fertilizer demand while maintaining soybean productivity in black soil regions.

## 5 Conclusions

The results of two-year field experiments revealed that combination of biochar with straw significantly enhanced soil hydrothermal conditions and increased total nitrogen (TN), total phosphorus (TP), total potassium (TK), and soil organic carbon (SOC) levels compared to straw alone. The combined application increased the amount of available nutrients for crop uptake, increased the chlorophyll content, photosynthesis, accumulation of photosynthates in soybeans and ultimately increased crop water use efficiency and nitrogen fertilizer use efficiency. Notably, a 20% reduction in nitrogen fertilizer did not diminish in soybean yields with the combined application of biochar and straw, whereas a 40% reduction did lead to a yield reduction. Therefore, the combined application of straw and biochar combined with a 20% reduction in nitrogen (SBN2) is recommended to improve the hydrothermal and nutrient status of the soil to maintain soybean yield. This study provides valuable insights for developing more effective and sustainable agricultural strategies for straw, biochar and nitrogen fertilizer management in black soil regions. In the future, it crucial to conduct long-term monitoring and comprehensive evaluation of the impacts of combined straw and biochar in agricultural fields.

## Data Availability

The original contributions presented in the study are included in the article/supplementary material. Further inquiries can be directed to the corresponding authors.
